# Ion transport regulation by P2Y receptors, protein kinase C and phosphatidylinositol 3-kinase within the semicircular canal duct epithelium

**DOI:** 10.1186/1756-0500-3-100

**Published:** 2010-04-14

**Authors:** Satyanarayana R Pondugula, Nithya N Raveendran, Daniel C Marcus

**Affiliations:** 1 Department of Anatomy and Physiology, Kansas State University, Manhattan, Kansas 66506, USA

## Abstract

**Background:**

The ionic composition of the luminal fluid in the vestibular labyrinth is maintained within tight limits by the many types of epithelial cells bounding the lumen. Regulatory mechanisms include systemic, paracrine and autocrine hormones along with their associated intracellular signal pathways. The epithelium lining the semicircular canal duct (SCCD) is a tissue that is known to absorb sodium and calcium and to secrete chloride.

**Findings:**

Transport function was assessed by measurements of short circuit current (*I*_*sc*_) and gene transcript expression was evaluated by microarray. Neither ATP nor UTP (100 microM) on the apical side of the epithelium had any effect on *I*_*sc*_. By contrast, basolateral ATP transiently increased *I*_*sc *_and transepithelial resistance dropped significantly after basolateral ATP and UTP. P2Y2 was the sole UTP-sensitive purinergic receptor expressed. *I*_*sc *_was reduced by 42%, 50% and 63% after knockdown of α-ENaC, stimulation of PKC and inhibition of PI3-K, while the latter two increased the transepithelial resistance. PKCdelta, PKCgamma and PI3-K were found to be expressed.

**Conclusions:**

These observations demonstrate that ion transport by the SCCD is regulated by P2Y2 purinergic receptors on the basolateral membrane that may respond to systemic or local agonists, such as ATP and/or UTP. The sodium absorption from endolymph mediated by ENaC in SCCD is regulated by signal pathways that include the kinases PKC and PI3-K. These three newly-identified regulatory components may prove to be valuable drug targets in the control of pathologic vestibular conditions involving dysfunction of transport homeostasis in the ear, such as Meniere's disease.

## Introduction

The transduction of linear and rotational acceleration into the sense of balance is driven and dependent on the ion composition of the luminal fluid, endolymph, of the vestibular labyrinth [1]. We have shown that the semicircular canal duct (SCCD) epithelium contributes to the homeostasis of endolymph by absorption of Na^+^, secretion of Cl^- ^and absorption of Ca^2+ ^[2-4]. These processes are known to be controlled by glucocorticoids, β-adrenergic agonists and vitamin D, respectively [2-4]. The present study augments previous investigations by reporting data that show the involvement of protein kinase C (PKC) and phosphatidylinositol 3-kinase (PI3-K) in the regulation of Na^+ ^transport by the SCCD and the regulation of transport and/or epithelial barrier function by the P2Y2 purinergic receptor.

Na^+ ^absorption is mediated by Na^+ ^entry from endolymph into the SCCD cytosol via apical epithelial Na^+ ^channels (ENaC) and removal of Na^+ ^from the cytosol across the basolateral membrane by the "Na^+^-pump", Na^+^, K^+^-ATPase [2]. The K^+ ^brought into the cell across the basolateral membrane by the Na^+^-pump recirculates passively through basolateral K^+ ^channels [2]. The classical ENaC is highly selective for Na^+ ^and is composed of α, β and γ subunits [5]. It has been found in mammals that expression of the three ENaC subunits undergo a tissue-specific non-coordinated regulation depending on the physiological context and this can occur at both the mRNA and protein levels [6]. In the current study, we have tested the specific involvement of α-ENaC in Na^+ ^absorption by SCCD by knockdown of gene expression with RNA interference.

The level of ENaC activity is regulated by a large number of factors, including complex signal pathways, such as protein kinase C (PKC) and phosphatidylinositol 3-kinase (PI3-K) signal pathways, which control the number of channels residing in the plasma membrane [7]. Protein turnover in the membrane is relatively rapid and signals controlling the insertion and removal of channels exerts powerful control over the rate of Na^+ ^absorption. The fractional open time of the channels is also regulated by PKC signaling pathways and phosphoinositide signal pathways that include activation of PI3-K [8-11]. The putative participation of PKC and phosphoinositide signaling in SCCD Na^+ ^absorption is tested in the current investigation by electrophysiology and gene array.

Purinergic signaling via receptors of the P2Y family constitutes an additional pathway that is known to regulate ENaC-mediated Na^+ ^absorption and Cl^- ^transport in other epithelia [12-15]. Experiments are described here that demonstrate the involvement of P2Y receptors in regulating transepithelial transport properties of SCCD, determine the location of the receptors and identify the candidate isoforms.

## Results and Discussion

Primary cultures of rat SCCD were tested for 1) regulation of ion transport by purinergic signaling, 2) participation of α-ENaC subunits in Na^+ ^absorption, 3) regulation of Na^+ ^absorption by a phosphoinositide signal pathway and 4) by a protein kinase C pathway.

### Array analysis of regulatory genes

Transcript analysis of genes investigated in this study are shown in Table S1 [Additional file 1]. Of the three UTP-sensitive receptors, only P2Y2 was present while P2Y4 and P2Y6 were absent and only P2Y2 was affected by dexamethasone treatment (known to regulate ENaC-mediated Na^+ ^transport) with a small decrease in expression. Expression results for the ionotropic P2X receptors were not consistent across all probe sets. There were probes for six isoforms of protein kinase C (PKC), and only PKCdelta and PKCgamma were present and there were no significant changes of expression in the presence of dexamethasone [Additional file 1]. PI3-K was found to be present and there was no significant change of expression with dexamethasone.

### Purinergic regulation of ion transport

**Figure 1:**
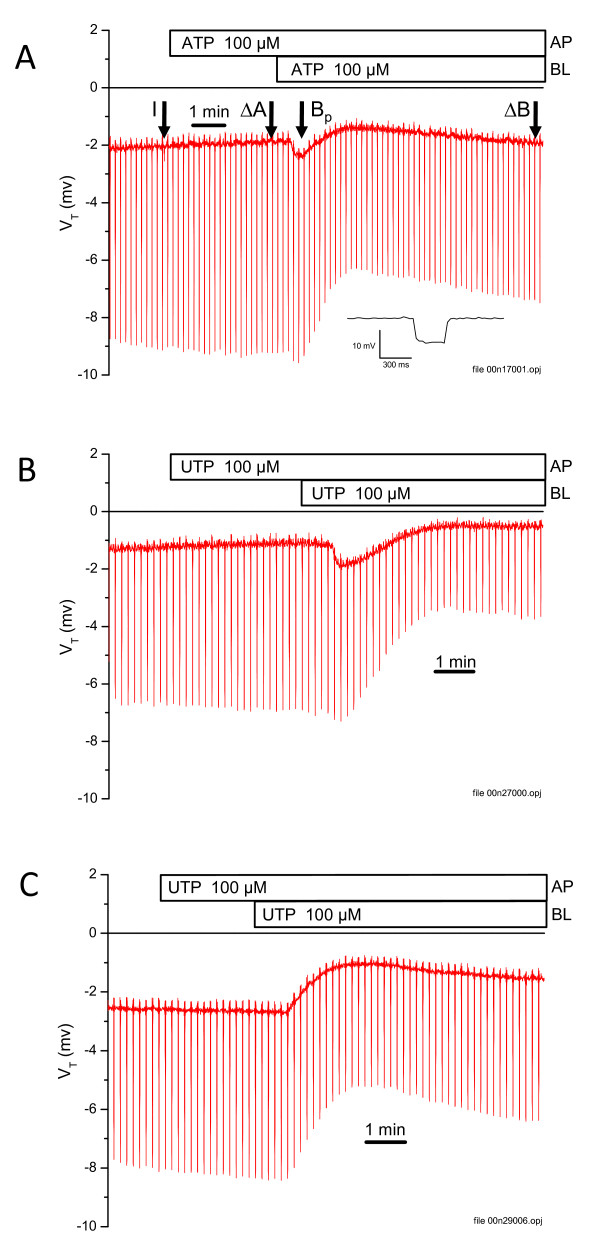
**Effects of purinergic agonists on short circuit current of SCCD epithelium**. Representative recordings of apical (AP) and basolateral (BL) addition of purinergic agonists to SCCD epithelia. The continuous line is the open-circuit transepithelial voltage (V_T_) and the pulses are the voltage response to current pulses. A) ATP was added to the apical or basolateral chamber at the times indicated by the bars at the top of the figure. A single pulse is shown with an expanded time scale in the insert for clarity and shows no time-dependent response. Vertical arrows indicate where readings were taken for the initial (I) value, change after apical (ΔA) agonist, peak basolateral (B_p_) and change after basolateral (ΔB) agonist for tabulation in Table 2. B) UTP was added to the apical or basolateral chamber at the times indicated by the bars at the top of the figure; example of recording with transient increase in V_T _after basolateral addition of UTP. C) Similar to recording in B, but an example of a recording without transient increase in V_T _after basolateral addition of UTP.

Many epithelia express one or more purinergic receptors that are inserted into the apical and/or basolateral cell membranes and anion or cation transport is often increased or decreased by activation of these receptors [16-21]. The P2 receptor agonists ATP and UTP had no effects on the transepithelial voltage (V_T_), resistance (R_T_) and equivalent short-circuit current (*Isc*) at the apical membrane, but they both exerted complex control from the basolateral side (Figure 1a; Table S2) [Additional file 2].

Basolateral ATP (100 μM) caused biphasic changes in 3 out of 4 epithelia (Figure 1a). Initially, there were statistically significant peak increases in V_T_. The R_T _did not change over this short interval (about 30 - 120 s after addition of ATP). The resistance dropped significantly over the next ~60 s in all epithelia (n = 4). That drop in resistance was accompanied by a decrease in V_T _from the peak, while *I*_*sc *_did not change, suggesting that the drop in V_T _was caused by the drop in R_T_.

Basolateral UTP (100 μM) yielded similar results with a full bi-phasic response of V_T _(Figure 1b) in one of three epithelia and all three epithelia exhibited the sustained decrease in V_T _and R_T _(Figure 1c; Table S2) [Additional file 2]. The resistance dropped significantly over ~2 min in all three epithelia. That drop in resistance was accompanied by a decrease of V_T _but no change in *I*_*sc*_, suggesting that the drop in V_T _was caused by the drop in R_T_, as for basolateral ATP.

ATP can activate most ionotropic P2X and P2Y family receptors. However, UTP is potent at only 3 of the P2Y receptors (P2Y2, P2Y4 and P2Y6) and none of the P2X receptors [16]. Our observations of transient stimulation of *I*_*sc *_and sustained decrease of R_T _along with the gene array results, suggest a dual signal pathway from basolateral P2Y2 receptors; one pathway controlling transepithelial ion transport and the other controlling the paracellular pathway. The results are in line with observations of UTP-stimulated Cl^- ^secretion in endometrial gland epithelial cells [15], but do not rule out a transient stimulation of Na^+ ^absorption. UTP-activated P2Y receptors have been shown to control paracellular tight junction pathways by decreasing the conductance in rabbit airway epithelium, opposite to the effect found here [22].

### Effect of α-ENaC siRNA on dexamethasone stimulated Na^+ ^absorption

**Figure 2:**
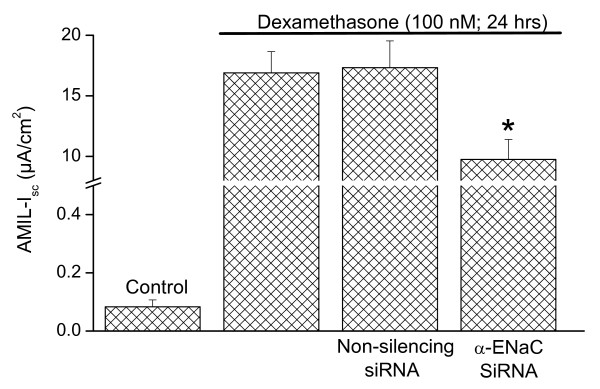
**Effect of RNA interference on short circuit current of SCCD epithelium**. Effect of α-ENaC siRNA on dexamethasone stimulated Na^+ ^transport: Epithelia were incubated with dexamethasone (100 nM) for 24 hr concurrently with transfection. Amiloride-sensitive short circuit current, AMIL-*I*_*sc*_, was measured from both transfected and non-transfected monolayers as a function of net Na^+ ^transport. α-ENaC siRNA (anti-sense) but not non-silencing siRNA (sense) inhibits dexamethasone-stimulated Na^+ ^transport. "Control" epithelia were not incubated with dexamethasone. Data are mean ± SEM, n = 3 - 10. P < 0.05 (*) compared to AMIL-*I*_*sc *_with dexamethasone alone. Note the break in the vertical axis.

Dexamethasone upregulates the expression levels of α-ENaC transcript and total protein in SCCD [23]. This result is consistent with α-ENaC being a limiting constituent that acts as a chaperone for the other subunits of ENaC in trafficking to the apical membrane [24]. We found (Figure 2) that α-ENaC siRNA (Table S3) [Additional file 3] significantly reduced the amiloride-sensitive *I*_*sc *_by 42% after incubation with dexamethasone (100 nM for 24 hr), whereas transfection with the non-silencing control siRNA (Table S3) [Additional file 3] changed the amiloride-sensitive *I*_*sc *_insignificantly (-2.5%). The specificity of the result is validated by the functional controls (transfection agent and non-silencing control siRNA) employed in the measurements of *I*_*sc*_. Amiloride is commonly used as a tool to block epithelial Na^+ ^transport mediated by ENaC [2].

### Effect of PKC activation and PI3-K inhibition on dexamethasone-stimulated Na^+ ^absorption

SCCD were incubated with dexamethasone (100 nM for 24 hr) in the presence or absence of the PKC activator phorbol-12-myristate-13-acetate (PMA; 100 nM, 24 h), its inactive analog 4-alpha-PDD (100 nM, 24 h) or the PI3-K inhibitors wortmannin (100 nM, 9 h) or LY294002 (20 μM, 9 h), or its inactive analog LY 303511 (20 μM, 9 h). Both PMA and the PI3-K inhibitors were used at concentrations that have been reported to alter Na^+ ^transport in other epithelia [9,11,25,26].

**Figure 3:**
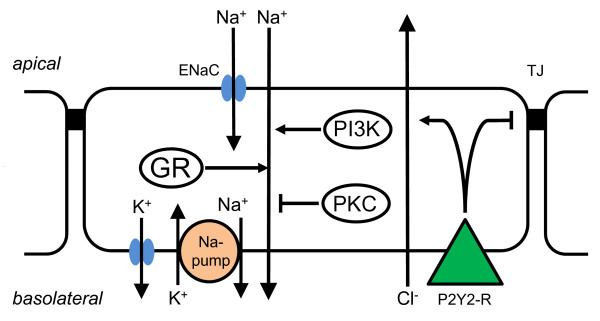
**Proposed cell model for P2Y, PKC and PI3-K regulation of ion transport by SCCD epithelium**. Na^+ ^is absorbed from endolymph by entry into the cytosol across the apical membrane via the epithelial Na^+ ^channel (ENaC). Na^+ ^is removed from the cytosol across the basolateral membrane by the Na^+^, K^+^-ATPase (Na^+^-pump); K^+ ^entering the cell on the Na^+^, K^+^-ATPase recycles across the basolateral membrane via K^+ ^channels. Activity of this Na^+ ^absorptive mechanism is stimulated by activation of the glucocorticoid receptor (GR), by activity of phosphoinositide-3 kinase (PI3-K) and inhibited by activity of protein kinase C (PKC). Activation of the P2Y2 receptor (P2Y2-R) in the basolateral membrane reduces the barrier to ions of the tight junctions (TJ) and stimulates Cl^- ^secretion.

Long-term exposure to PMA, but not 4-alpha-PDD, significantly reduced *I*_*sc *_by 49.7% and increased R_T _by 28.3% (Table S4) [Additional file 4]. Similarly, LY 294002, but not LY 303511, significantly reduced *I*_*sc *_by 62.5% and increased R_T _by 179% (Table S4) [Additional file 4]. Further, Wortmannin also inhibited *I*_*sc *_by 21.8% (Table S4) [Additional file 4]. The effects of PMA and LY 294002 on dexamethasone stimulated *I*_*sc*_, and on R_T _are consistent with positive regulation by PI3-K and negative regulation by PKC of glucocorticoid-stimulated Na^+ ^absorption by SCCD epithelium (Figure 3).

Both PKC and PI3-K signaling pathways regulate corticosteroid-stimulated Na^+ ^transport in several epithelial cells and expression systems via acute regulation of the open probability and rapid insertion and removal of ENaC in the cell membrane [8-11] and also the long-term expression level of ENaC in the apical membrane via regulation of ubiquitin-mediated degradation [27,28].

## Conclusions

The present study augments previous reports of ion transport systems that are expressed and characterized in SCCD epithelium, including sodium absorption and chloride secretion. We showed here that sodium absorption from endolymph mediated by ENaC in SCCD is regulated by signal pathways that include the kinases PKC and PI3-K (Figure 3). Further, purinergic receptors control both active ion transport and the integrity of the epithelial barrier. These kinases and the purinergic receptor(s) may prove to be valuable drug targets in the control of pathologic vestibular conditions such as Meniere's disease that involve dysfunction of transport homeostasis in the ear.

## Materials and methods

### Primary cultures of SCCD epithelium

Primary cultures from the semicircular canals of neonatal (3-5 days) Wistar rats were grown on 6.5 mm diameter Transwell permeable supports as described previously [2,3,23] (see Table S5 in Additional File 5 "Additional methods" for additional details of methods).

### Materials

Amiloride (#A-7410, Sigma), Phorbol-12-myristate-13-acetate (PMA; #524400, Calbiochem), 4-alpha-phorbol-12, 13-didecanoate (4 alpha-PDD; #524394, Calbiochem), 2-(4-Morpholinyl)-8-phenyl-4H-1-benzopyran-4-one (LY 294002; #L-9908, Sigma) and wortmannin (#W1628, Sigma) were dissolved in DMSO. Cyclodextrin-encapsulated dexamethasone (#D-2915, Sigma), 2-piperazinyl-8-phenyl-4H-1-benzopyran-4-one (LY 303511; #L-2786, Sigma) were dissolved in water. The purinergic agonist UTP (#U6625, Sigma) was dissolved directly in the physiological saline (below).

### Electrophysiological measurements

Epithelia were bathed in symmetrical bicarbonate-buffered physiological saline and the transepithelial voltage (V_T_), resistance (R_T_) and equivalent short-circuit current (*Isc*) were measured as before [2] (see additional File 5). Amiloride-sensitive *I*_*sc *_(AMIL - *I*_*sc*_), which corresponds to the net transepithelial Na^+ ^absorption, was measured for each condition studied. Each experimental series was performed on paired cultures from a single batch of dissociated canals.

### siRNA

Rat α-ENaC siRNA (four sets of sequences given in Table S1 [Additional file 1; Additional file 3] [29]) were designed by Qiagen company based on an available rat α-ENaC cDNA nucleotide sequence (GenBank Accession NM_031548). Both α-ENaC and non-silencing fluorescein-labeled control siRNA (#1022079, Qiagen) were reconstituted in siRNA Suspension Buffer (#301699, Qiagen).

### Analysis of electrophysiologic data

Data are presented as mean values ± SEM from n observations. Data in Figure 1 were smoothed with a 15-point FFT smoothing algorithm in Origin software (OriginLab, Northampton, MA). An expanded, unfiltered pulse is shown in the inset to Figure 1a. Student's *t*-test was used to determine statistical significance of paired (ATP and UTP experiments) and unpaired samples. Differences were considered significant for *P *< 0.05.

### Gene array analysis

Total RNA was extracted from control (n = 4) and dexamethasone-treated (n = 4) samples of SCCD primary cultures using RNeasy Micro Kit. The quality and quantity were determined as described previously [23]. Affymetrix microarrays (see additional File 5 for details) were used to examine the expression of the genes investigated here by electrophysiology. Our methodology conforms to the MIAME (Minimum Information about a Microarray Experiment) guidelines and details are deposited with the data in [GEO: GSE6197].

Statistical algorithms [detection, change call, signal log ratio (SLR)] were used to identify differential gene expression in control and experimental samples. Probe sets were assigned as either present (P), absent (A), or marginal (M) based on detection *p *values (P, *p *< 0.05; M, *p *= 0.05-0.065; A, *p *> 0.065). Fold change was calculated from the median of the SLRs and only considered physiologically significant when the fold change was ≥ 1.3.

## Competing interests

The authors declare that they have no competing interests.

## Authors' contributions

SRP carried out the study, including microdissection of tissues, primary cell cultures, quantitative analyses and contributed to writing the manuscript. NNR carried out the gene array analyses. DCM conceived of the study, participated in its design and coordination and contributed to writing the manuscript. Both authors read and approved the final manuscript.

## Supplementary Material

Additional file 1 **Table S1. Gene array of rat SCCD**. Presence call and fold-change of transcripts for purinergic receptors, protein kinase C and phosphatidylinositol-3 kinase in SCCD epithelium. Click here for file

Additional file 2 **Table S2. Effects of ATP & UTP on SCCD transport**. Effects of purinergic agonists on electrophysiology of ion transport. Click here for file

Additional file 3 **Table S3. siRNA for alpha-ENaC**. Rat α-ENaC siRNA sequences used to knock down the functional expression of the α subunit of ENaC. Click here for file

Additional file 4 **Table S4. PKC and PI3-K regulation of Isc**. Effects of PKC activator and PI3-K inhibitors on Isc and RT. Click here for file

Additional file 5 **Table S5. Gene chip quality metrics**. Quality metrics of Affymetrix gene chips. Click here for file
