# Supplementary material for: Ion transport regulation by P2Y receptors, protein kinase C and phosphatidylinositol 3-kinase within the semicircular canal duct epithelium
Source: BMC Res Notes. 2010 Apr 14;3:100. doi: 10.1186/1756-0500-3-100 (PMC2862037; doi:10.1186/1756-0500-3-100)
Supplement: Additional file 1 — Table S1. Gene array of rat SCCD. Presence call and fold-change of transcripts for purinergic receptors, protein kinase C and phosphatidylinositol-3 kinase in SCCD epithelium. [file 1756-0500-3-100-S1.PDF]

Table 1 – Gene array of rat SCCD. Presence call and fold-change of transcripts for purinergic receptors, protein kinase C and phosphatidylinositol-3 kinase in SCCD epithelium.

			Present (P) / Absent (A)		
Affymetrix. Number	Gene Name	Product	- Dex	+ Dex	Fold-change
1370606_at	P2ry1	P2Y1 Receptor	A	A	---
1368940_at	P2ry2	P2Y2 Receptor	4P	4P	-1.32*
1369812_at	P2ry4	P2Y4 Receptor	A	A	---
1368754_at	P2ry6	P2Y6 Receptor	A	A	---
1368740_at	P2rxl1	P2Xl1 Receptor	A	A	---
1369178_a_at	P2rx1	P2X1 Receptor	A	A	---
1387578_a_at	P2rx2	P2X2 Receptor	3P	4P	1.07
1369474_a_at	P2rx2	P2X2 Receptor	3P	4P	1.07
1369475_x_at	P2rx2	P2X2 Receptor	A	A	---
1369475_x_at	P2rx2	P2X2 Receptor	A	A	---
1388077_a_at	P2rx3	P2X3 Receptor	A	A	---
1368667_at	P2rx3	P2X3 Receptor	A	A	---
1373775_at	P2rx4	P2X4 Receptor	4P	4P	-1.07
1369743_a_at	P2rx4	P2X4 Receptor	4P	4P	1.23
1369674_at	P2rx5	P2X5 Receptor	3P	3P	1.07
1369673_at	P2rx5	P2X5 Receptor	A	A	---
1387718_at	P2rx7	P2X7 Receptor	A	A	---
1370585_a_at	Prkcb1	Protein kinase C beta	A	A	---
1368240_a_at	Prkcb1	Protein kinase C beta	A	A	---
1387114_at	Prkcd	protein kinase C, delta	P	P	1.07
1369830_at	Prkch	protein kinase C, eta	A	A	---
1369089_at	Prkcc	protein kinase C, gamma	P	P	-1.23
1369620_at	Prkci	protein kinase C, iota	A	A	---
1370197_a_at	Prkcz	protein kinase C, zeta	A	A	---
1369655_at	Pik3c3	phosphatidylinositol 3-kinase	4P	4P	1.23

*, physiologically-significant fold change in expression after dexamethasone (Dex). #P, number of gene chips out of 4 per condition that registered P; minus sign for fold change indicates down-regulation. SCCD, semicircular canal duct epithelium. Fold change was calculated from the median of the SLRs and only considered physiologically significant when the fold change was greater than or equal to 1.3. This analysis by SLR accounts for the differences in individual hybridization efficiencies at the individual probe pair level.
