# Supplementary material for: Ion transport regulation by P2Y receptors, protein kinase C and phosphatidylinositol 3-kinase within the semicircular canal duct epithelium
Source: BMC Res Notes. 2010 Apr 14;3:100. doi: 10.1186/1756-0500-3-100 (PMC2862037; doi:10.1186/1756-0500-3-100)
Supplement: Additional file 2 — Table S2. Effects of ATP & UTP on SCCD transport. Effects of purinergic agonists on electrophysiology of ion transport. [file 1756-0500-3-100-S2.PDF]

Table 2 - Effects of ATP & UTP on SCCD transport. Effects of purinergic agonists on electrophysiology of ion transport.

	Initial			Δ Apical Membrane			Δ Basolateral Peak			Δ Basolateral Steady State		
	VT (mV)	RT (Ω-cm^2)	Isc (μA/cm^2)	VT (mV)	RT (Ω-cm^2)	Isc (μA/cm^2)	VT (mV)	RT (Ω-cm^2)	Isc (μA/cm^2)	VT (mV)	RT (Ω-cm^2)	Isc (μA/cm^2)
ATP (100 μM) n=4	-1.49 ± 0.22	1752 ± 289	-0.87 ± 0.07	-0.05 ± 0.06 ns	17 ± 18 ns	0.04 ± 0.03 ns	0.51 ± 0.04 *	29 ± 21 ns	0.30 ± 0.09 *	0.87 ± 0.11 *	620 ± 118 *	0.24 ± 0.24 ns
UTP (100 μM) n=3	-2.10 ± 0.43	1846 ± 29	-1.13 ± 0.23	0 ± 0 ns	0 ± 0 ns	0 ± 0 ns	0.70 (n=1)	0 (n=1)	7.07 (n=1)	1.25 ± 0.32 *	623 ± 129 *	2.18 ± 0.83 ns

*, significant change—paired t-test; ns, insignificant change. SCCD, semicircular canal duct epithelium. Times at which data were taken for this table are illustrated in Figure 1a by vertical arrows. When no discernible change in parameters was observed in all experiments of a series, the table entry is "0".
