# Supplementary material for: Ion transport regulation by P2Y receptors, protein kinase C and phosphatidylinositol 3-kinase within the semicircular canal duct epithelium
Source: BMC Res Notes. 2010 Apr 14;3:100. doi: 10.1186/1756-0500-3-100 (PMC2862037; doi:10.1186/1756-0500-3-100)
Supplement: Additional file 3 — Table S3. siRNA for alpha-ENaC. Rat α-ENaC siRNA sequences used to knock down the functional expression of the α subunit of ENaC. [file 1756-0500-3-100-S3.PDF]

Table 3 - siRNA for alpha-ENaC. Rat α-ENaC siRNA sequences used to knock down the functional expression of the α subunit of ENaC.

Sets of DNA target sequences	Sense siRNA (5' – 3')	Antisense siRNA (5' – 3')
AAGCCCTGGGCAACTTCATCT	r(GCCCUGGGCAACUUCAUCU)d(TT)	r(AGAUGAAGUUGCCCAGGGC)d(TT)
AACTGTGCAACCAGAACAAAT	r(CUGUGCAACCAGAACAAAU)d(TT)	r(AUUUGUUCUGGUUGCACAG)d(TT)
AAACGGAGTTGCAAAGCTCAA	r(ACGGAGUUGCAAAGCUCAA)d(TT)	r(UUGAGCUUUGCAACUCCGU)d(TT)
AAGAAGCCCTGGGCAACTTCA	r(GAAGCCCUGGGCAACUUCA)d(TT)	r(UGAAGUUGCCCAGGGCUUC)d(TT)

α-ENaC siRNA sequences were designed by Qiagen based on the cDNA sequence of rat α ENaC (Gene Bank Accession No: NM 031548). [59]
