# Supplementary material for: Ion transport regulation by P2Y receptors, protein kinase C and phosphatidylinositol 3-kinase within the semicircular canal duct epithelium
Source: BMC Res Notes. 2010 Apr 14;3:100. doi: 10.1186/1756-0500-3-100 (PMC2862037; doi:10.1186/1756-0500-3-100)
Supplement: Additional file 4 — Table S4. PKC and PI3-K regulation of Isc. Effects of PKC activator and PI3-K inhibitors on Isc and RT. [file 1756-0500-3-100-S4.PDF]

Table 4 - PKC and PI3-K regulation of Isc. Effects of PKC activator and PI3-K inhibitors on *Isc* and R_T.

Condition	(n)	Amiloride-sensitive I_{sc} (µA/cm^2)	R_T (Kohm-cm^2)
DEX	(3)	11.9 ± 0.9	2.37 ± 0.13
DEX + PMA (100 nM; 24 hr)	(3)	6.0 ± 0.6*	3.04 ± 0.15*
DEX	(3)	11.6 ± 0.3	2.81 ± 0.26
DEX + 4-alpha-PDD (100 nM; 24 hr)	(3)	11.3 ± 0.5	3.01 ± 0.21
DEX	(3)	10.4 ± 0.7	1.48 ± 0.35
DEX + LY 294002 (20 µM; 9 hr)	(3)	4.0 ± 0.6*	4.13 ± 0.02*
DEX	(3)	11.6 ± 0.7	2.42 ± 0.17
DEX + LY 303115 (20 µM; 9 hr)	(3)	10.5 ± 0.4	2.60 ± 0.20
DEX	(3)	11.6 ± 0.3	2.81 ± 0.26
DEX + Wortmannin (100 nM; 9 hr)	(3)	9.6 ± 0.3*	2.35 ± 0.33

Response of amiloride (10 µM)-sensitive I_{sc} and transepithelial resistance (R_T) by SCCD epithelia to dexamethasone (DEX [100 nM; 24 hr]) treatment in the presence and absence of either PKC activator or PI3-K inhibitors. Each pair of control and treated epithelia cultures were taken from one batch of SCCD monolayers. R_T, transepithelial resistance. Values are means ± SEM; n, number of experiments in parenthesis. DEX, dexamethasone; PMA, Phorbol-12-myristate-13-acetate; 4-alpha-PDD, 4-alpha-phorbol-12, 13-didecanoate; LY 294002, 2-(4-Morpholinyl)-8-phenyl-4H-1-benzopyran-4-one; LY 303511, 2-piperazinyl-8-phenyl-4H-1-benzopyran-4-one. The unpaired *t*-test was used to compare DEX to DEX + PKC activator and DEX + PI3-K inhibitors. $*P < 0.05$. SCCD, semicircular canal duct epithelium.
