# Supplementary material for: Ion transport regulation by P2Y receptors, protein kinase C and phosphatidylinositol 3-kinase within the semicircular canal duct epithelium
Source: BMC Res Notes. 2010 Apr 14;3:100. doi: 10.1186/1756-0500-3-100 (PMC2862037; doi:10.1186/1756-0500-3-100)
Supplement: Additional file 5 — Table S5. Gene chip quality metrics. Quality metrics of Affymetrix gene chips. [file 1756-0500-3-100-S5.PDF]

Table 5. Quality metrics of Affymetrix gene chips (Affymetrix Rat RAE 230A and 230B; Affymetrix, Santa Clara, CA)**.**

	Average Background	Standard Deviation Background	Average Noise	Standard Deviation Noise	Average Raw Q	Average Scale Factor
Series A chips (n=4)	93.9	32.9	5.65	1.91	3.08	1.50
Series B chips (n=4)	87.8	30.9	4.40	1.66	2.96	5.33
